# Improved Understanding of Traumatic Complex Elbow Instability

**DOI:** 10.5435/JAAOSGlobal-D-23-00041

**Published:** 2023-09-22

**Authors:** John J. Heifner, Deana M. Mercer

**Affiliations:** From the Miami Orthopaedic Research Foundation, Miami, FL (Dr. Heifner), and the University of New Mexico Department of Orthopaedics and Rehabilitation, Albuquerque, NM (Dr. Mercer).

## Abstract

Recent advancements in surgical treatment have improved clinical results in complex traumatic elbow injury. There is increasing recognition that conservative treatment and inadequate surgical fixation carry high risk of substantial morbidity in many of these cases. Recent literature displays improved outcomes in complex elbow instability, in part, because of a more complete comprehension of the injury patterns and fixation methods. Prompt surgical management with stable internal fixation, which permits immediate postoperative mobilization, has been a consistent variable across the reports leading to more satisfactory outcomes. This applies to both acute and chronic cases.

Elbow dislocations are stratified into simple and complex. A simple elbow dislocation typically has no associated fractures or soft-tissue injury, including brachialis and collateral ligaments, and is stable after reduction, allowing early mobilization and return of nearly full function. Complex elbow injuries often have associated fractures in addition to soft-tissue injuries. The fractures may include the radial head, olecranon, and/or coronoid. For example, an elbow dislocation with a coronoid fracture may indicate a greater risk of instability.

Recent advancements in the understanding of injury patterns have led to improved surgical treatment algorithms and better clinical outcomes in complex elbow injury.^1^ Historically, surgeons were not eager to operate on these complex injuries because outcomes were poor. As our understanding of injury patterns and surgical fixation techniques has improved, surgery led to better outcomes. The indication for surgical treatment in complex elbow injuries is less controversial, but there is continued debate regarding which components of the injury need to be surgically addressed and which fixation options may be most effective for each injury component. There is increasing recognition that conservative treatment and inappropriate surgical fixation carry high risk of substantial morbidity in many of these cases.

In this review, we will define various patterns of complex elbow instability. Within each of these injury patterns, recent evidence will be presented for the mechanism of injury, patterns of disruption, and surgical management.

## Anatomy and Kinematics

Elbow stability is primarily dependent on the synergistic constraints of the osseous, muscular, and ligamentous structures. The articular surfaces of the elbow are the ulnohumeral joint and radiocapitellar joint, which allow motion as a hinge in the sagittal plane and rotation in the axial plane, respectively. The coronoid and radial head provide an anterior buttress, preventing posterior dislocation of the forearm. The olecranon and proximal ulna prevent anterior dislocation of the forearm. The ulnohumeral joint is highly congruent and inherently stable, and the radial head stabilizes against valgus forces.

Static constraints include the medial and lateral collateral ligaments and joint capsule. Each collateral ligament contains distinct components, which collectively provide multiplanar restraint. The medial collateral ligament (MCL) or ulnar collateral ligament consists of an anterior, posterior, and transverse bundle and provides stability against valgus stress. The proportion of valgus restraint provided by the MCL differs with the degree of elbow flexion, with the highest proportion being provided at 90° of elbow flexion. The anterior bundle inserts onto the sublime tubercle of the coronoid and provides restraint against posteromedial rotatory instability (PMRI). This bony-ligamentous junction is important to consider when evaluating coronoid fractures. The lateral collateral ligament (LCL) comprises a radial, ulnar, annular, and accessory bundle. This complex maintains tension throughout the elbow flexion-extension arc and thus is the primary contributor to multidirectional elbow stability. The LCL provides support for the radiocapitellar, ulnohumeral, and proximal radioulnar joints, specifically restraining against posterolateral rotatory and varus stress. With its insertions on the anterior and posterior radial notch, the annular ligament envelops the radial head and thus stabilizes the radioulnar relationship within the proximal radioulnar joint.

Elbow trauma due to axial load being transmitted proximally through the forearm can produce concomitant disruption of the interosseous ligament complex (IOLC), furthering the severity of elbow instability. The IOLC has three distinct components—the proximal oblique ligaments, the central band, and the distal oblique ligaments. The ligamentous fiber orientation of these three separate distinct structures defines their function. With fibers that course from the proximal ulna to the distal radius, the distal and proximal oblique ligaments prevent distal migration of the radius in relation to the ulna. The central band fibers course from the proximal radius to the distal ulna, which prevents proximal migration of the radius in relation to the ulna. The fibers of the central band are approximately 24° in relation to the longitudinal axis of the forearm.^2^ The mechanism of force transmission by the central band from the proximal radius to the distal ulna is displayed in various injury patterns, including Galeazzi and Essex-Lopresti fractures. In addition, the central band maintains longitudinal stability of the forearm, which prevents distal ulnar migration and ulnar impaction.^3^ Injury to the IOLC in conjunction with complex elbow injury may lead to more involved injury patterns and necessitate modified treatment algorithms.

## Common Injury Patterns

### Proximal Ulna Fracture-Dislocation

Fractures of the proximal ulna are classified based on the location of the fracture and the subsequent joint disruption. Ring et al^4^ provided clarity for the delineation of these injury patterns into transolecranon fracture-dislocation and Monteggia fractures of the proximal ulna. Transolecranon fractures occur within the greater sigmoid notch leading to disruption of ulnohumeral continuity, but they have an intact proximal radioulnar joint. Monteggia fractures occur within the proximal ulna and may have extension into the greater sigmoid notch. Notably, these injuries have disruption of the proximal radioulnar joint. Both injury patterns can present with discontinuity of the greater sigmoid notch, but the unique identifier is the state of the proximal radioulnar joint, which is disrupted in Monteggia fractures and intact in transolecranon fractures.

Monteggia fractures may present with anterior or posterior dislocation of the proximal radius. The Bado classification is the most commonly cited classification system of Monteggia fracture patterns. Injuries are classified based on the direction of dislocation of the proximal radius and, further, on the characteristics of the proximal ulna fracture. The two most common injuries are Type I, which is an anterior dislocation of the proximal radius, and Type II, which is a posterior dislocation of the proximal radius. Jupiter et al^5^ provided an expanded classification for posterior Monteggia fractures based on the location of the ulna fracture, in proximity to and inclusive of the coronoid. Historically, these cases demonstrated unsatisfactory outcomes. In aggregate, the most recent literature has displayed a trend toward improved outcomes in Monteggia cases with agreement that improved injury understanding and the use of stable fixation have been the major contributors.^6^

Similar to Monteggia fractures, transolecranon fracture-dislocations can span a wide range of traumatic force application. A recent systematic review compiled a sample of 105 transolecranon fracture cases, reporting coronoid fractures in 53%, radial head fractures in 17%, and both fractures in only 7% of these cases.^7^ Although these injuries have a complex fracture pattern, it was originally thought that they did not often have disruption of the collateral ligaments.^4,8^ Recent evidence suggests that ligamentous disruption may be more common than stated in historical reports. A retrospective follow-up of 35 transolecranon fractures reported concomitant LCL disruption in 11% (4/35) cases.^9^ Each of these cases had radial head and coronoid fractures in addition to the olecranon fracture, which implies a high-energy mechanism of injury. Thus, the authors recommended that surgeons be alerted to the potential for collateral ligament injury in transolecranon fracture cases that present with associated fractures. A recent biomechanical study quantified the allowable limits of inferior humerus translation after a simulated olecranon fracture.^10^ Based on the findings, the authors recommended a high degree of suspicion for LCL or MCL disruption if the humerus is inferiorly translated more than 3 mm and if translated more than 7.5 mm, there may be high potential for concomitant LCL and MCL disruption. The consistent evaluation of transolecranon fractures as an osseous disruption with spared ligamentous structures may need to be updated given these recent clinical and biomechanical findings. Collectively, the literature reports are encouraging because satisfactory results can be achieved after surgical management of all components of the injury, both bony and ligamentous, in these complex cases.^8,9^

**Figure 1:**
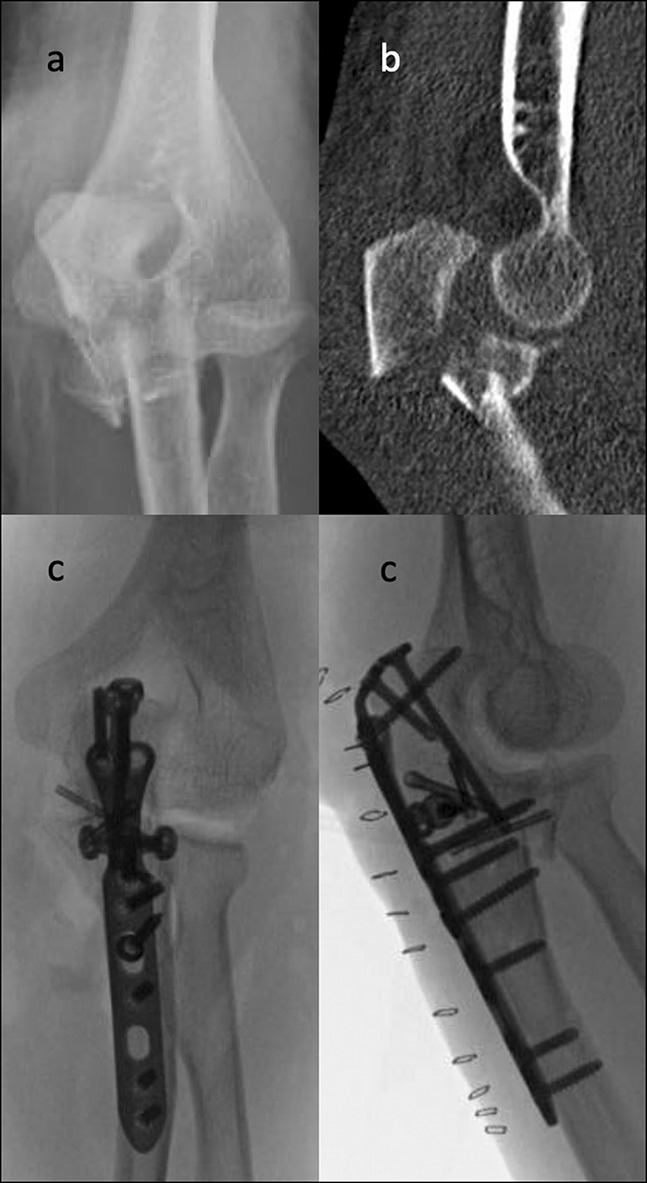
Radiology images (**A**) and CT scan (**B**) demonstrating transolecranon fracture-dislocation of the elbow. Radiographs demonstrating surgical fixation using a proximal ulna plate and screws (**C**).

Given the joint involvement in transolecranon fracture-dislocations and Monteggia fractures, the specific techniques of surgical fixation differ. Although Monteggia cases may have disruption of the greater sigmoid notch, the surgeon must reapproximate the proximal ulna to anatomically restore the proximal radioulnar joint. Conversely, in transolecranon cases, which have an intact proximal radioulnar joint, the surgeon must reapproximate the articular surface of the greater sigmoid notch (Figure 1). Fixation of the radial head and coronoid is preferable in cases with an adequately sized coronoid and radial head fragments that are amenable to reduction and fixation. In cases of complex comminuted radial head fracture, a radial head arthroplasty may be indicated for improved function.

### Terrible Triad Injury

A terrible triad injury of the elbow is defined as an elbow dislocation with radial head fracture, coronoid fracture, and often a LCL disruption. The term ‘terrible triad’ was popularized because of the complexity of surgical management and the consistently poor outcomes reported in these cases. Recent evidence has demonstrated various mechanistic scenarios which culminate in this injury pattern. Classically described as a posterolateral external rotation injury—which is the most common mechanism—these injuries less commonly occur because of posteromedial external rotation and posteromedial internal rotation.^11^ The common component of these mechanisms is the rotatory force which fractures both anterior osseous structures. Greater degrees of external rotation result in smaller fractured fragments of the radial head and coronoid, and lesser degrees of external rotation result in larger fractured fragments of the radial head and coronoid. This inverse relationship between degree of external rotation and size of the fractured fragments is because of the greater clearance between the proximal radius and ulna and the distal humerus in more forceful injury patterns. Furthermore, the external rotation force in posterolateral dislocations results in disruption of the LCL by a stripping mechanism.^11^ For posteromedial dislocations—due to external and internal rotation—the LCL mechanism of injury is distraction due to the varus force, which produces the posteromedial translation of the proximal radius and ulna. Evidence strongly supports surgical repair of the LCL to provide an important contribution to the radial-sided stability. This practice has been an integral component of recent surgical algorithms that have yielded improved outcomes.^12,13^

**Figure 2:**
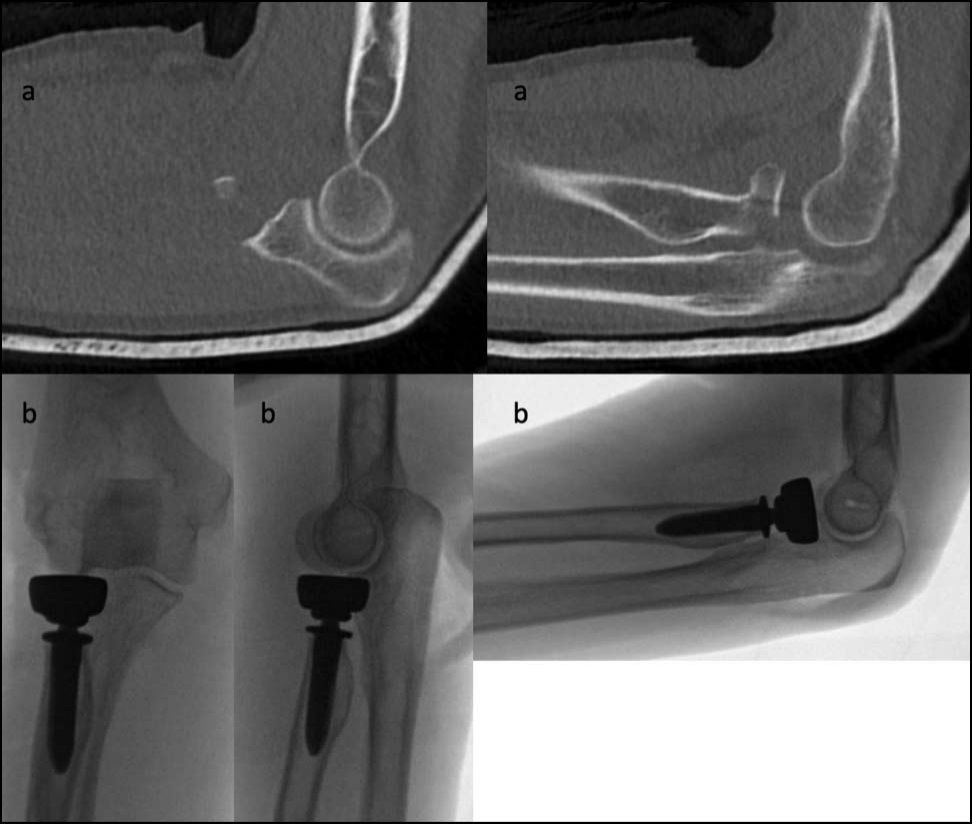
Postreduction CT scans demonstrating terrible triad injury of the elbow with radial head and coronoid fracture (**A**). Radiographs demonstrating surgical fixation using a radial head prosthesis (**B**).

The stabilizing effect of the coronoid and radial head has been well-described.^1^ There is agreement on the need to acutely stabilize the elbow with internal fixation to hasten mobilization (Figure 2). However, there remains debate on specifics such as which disrupted elements should be addressed and the determination of appropriate techniques. While a select few triad injuries may be indicated for conservative treatment, surgical restoration of the buttressing support of the radial head and coronoid maximizes stability and function.

The need for coronoid fixation is predicated on the size of the fractured fragment and the presence of a reconstructed or replaced radial head.^13-15^ Coronoid fractures are classified by the percentage of the coronoid that is fractured and the location of the fracture and concomitant injury.^16,17^ These factors contribute to the likelihood of instability. Recent biomechanical^15^ and clinical^13^ evidence suggests that coronoid fracture fragments measuring approximately 50% or less of the whole—Regan-Morrey Type I and II—can remain unfixed in the presence of a repaired LCL and reconstructed or replaced radial head in triad injuries. In the presence of larger fragments of the coronoid, stable internal fixation is requisite to stabilize the elbow and restore the anterior buttress.

There is consistent evidence for the importance of the stabilizing support of the proximal radius.^18^ Radial head excision portends unsatisfactory outcomes in terrible triad injuries and is only performed within narrow indications. However, the subsequent choice between open reduction and internal fixation (ORIF) and radial head arthroplasty (RHA) continues to be controversial, and the literature is rife with conflicting evidence. Chen et al^19^ conducted a systematic review comparing ORIF (N = 51) and RHA (N = 64) in triad injury. Their results demonstrated markedly better range of elbow motion with fewer complications for RHA across a follow-up period ranging from 24 to 41 months. Klug et al^20^ compared ORIF (N = 32) with RHA (N = 26) in triad cases at a mean follow-up of 4.5 years. ORIF cases had markedly higher scores across multiple outcome metrics with lower rates of complication and revision surgery.

In conflict with the reports that describe superiority of either ORIF or RHA, others have concluded comparable outcomes between these treatment methods. A recent systematic review compared ORIF with RHA across 210 triad cases, concluding no differences between the treatment methods for Mayo Elbow Performance Score (MEPS), elbow arc of motion, and rate of revision surgery.^21^ There does seem to be a trend toward RHA providing more favorable outcomes for short to mid-term follow-up compared with ORIF. Some would contend that the concern with RHA is not outcomes in the short to mid-term, but those in the long term, especially considering the frequency of these injuries occurring in young to middle-aged adults.

Radial head prosthesis design has been identified as an important factor in determining the success of the arthroplasty surgery.^22^ Prosthesis characteristics have been biomechanically evaluated in simulated triad injury.^23^ The results demonstrated that anatomic radial head design and monopolarity contributed to greater radiocapitellar stability. RHA has seen a recent evolution in prosthesis design, which may contribute to improved durability and satisfactory outcomes in the long term.^22,24^ The design characteristics of fixed-stem implants are highly variable, which likely contributes to the conflicting outcomes which have been reported for these implants.^22^ A recent systematic review by Said et al^25^ demonstrated similar clinical outcomes between bipolar and monopolar prostheses. By contrast, systematic reviews by Chen et al and Vannabouathong et al reported superior radiological findings for monopolar compared with bipolar prostheses.^22,26^ The biomechanical work by Moungondo et al demonstrated a reduced radiocapitellar contact area for monopolar and bipolar prostheses compared with the native joint.^27^ Furthermore, bipolar designs had a markedly reduced contact area in neutral forearm rotation because of subluxation.

In aggregate, the recent literature for radial head treatment in terrible triad injury has demonstrated favorable outcomes. This improvement in outcomes is encouraging and supports the importance of surgical treatment. An integral component of this improvement is the more rapid commencement of active motion, which is predicated on stable internal fixation.^12^ Prolonged immobilization after unstable complex elbow injury has consistently been a harbinger for poor outcomes and increased morbidity.

### Anteromedial Coronoid Fracture

PMRI occurs because of fracture of the anteromedial facet of the coronoid. The fracture mechanism is an axial force with varus torque that transmits proximally to the elbow causing the anteromedial coronoid to collide with the trochlea. Posteromedial dislocation of the ulnohumeral joint can occur after anteromedial coronoid fracture (AMCF). There is a lack of consistency in injury presentation because of the magnitude of applied force, inherent morphology, and limitations in diagnostic capability.^28^ The rotatory component of the applied force can cause additional soft-tissue disruption of the LCL and MCL with potential disruption of both the anterior (aMCL) and posterior (pMCL) bands of the MCL.^28,29^ Of note, radial head fracture is rarely seen in this injury pattern because of the mechanics of the applied load. This injury pattern is complex because of the structures that are disrupted, the difficulty in recognition of the injury pattern, and the uncertainty for which elements require surgical management. Further, missed, or delayed diagnoses of these fractures portend substantial morbidity.

In both posterolateral rotatory instability (PLRI) and PMRI, the ulnohumeral joint is subluxated. However, the lack of spontaneous joint reduction is a differentiator of these pathologies. In PMRI, the elbow is often persistently subluxated, whereas in PLRI, the elbow spontaneously reduces. The lack of joint concentricity in PMRI is because of the repetitive varus loads on the elbow during activities of daily living, which stresses the anteromedial coronoid, creating pathologic joint contact pressures in the presence of AMCF.^30^ Markolf et al^31^ determined that up to 93% of applied axial loads are transmitted through the coronoid under varus stress. Coronoid incongruity has demonstrated increased point loading at the ulnohumeral articular surface, which predisposes to degenerative disease. Hwang et al^32^ studied ulnohumeral joint contact pressures in the native elbow and in the setting of PMRI. Testing was done in specimens with AMCF, with additional LCL disruption, and with additional pMCL disruption. The authors concluded that PMRI cases with ulnohumeral subluxation likely had disruption of the pMCL. These findings are contrasted with the biomechanical work by Bellato et al who concluded that LCL disruption was necessary to produce ulnohumeral joint subluxation in the presence of AMCF and a disrupted pMCL. Bellato et al^33^ tested specimens with AMCF, with additional LCL disruption, and with additional pMCL disruption. Hwang et al^32^ reported that joint contact pressures were markedly higher in isolated AMCFs and in AMCFs with LCL disruption when compared with the native joint. These findings are in agreement with Bellato et al^33^ who reported on elevation in joint contact pressures with AMCF and LCL disruption. Collectively, these authors recommend repair of the coronoid and LCL, which can provide near-native joint pressures and thus reduce the risk of degenerative articular disease.

Factors that contribute to the presence and degree of instability include the size and location of the fractured component(s). The expanded coronoid fracture classification by O'Driscoll provided guidance to facilitate identification of fracture patterns and the potential for concomitant injury that may not be readily apparent.^1^ A recent systematic review on AMCF stratified outcomes based on the O'Driscoll classification.^34^ Across the sample of 114 cases, 89% were surgically treated with a mean MEPS of 91.5. Type I fractures had a mean MEPS of 97 (N = 5), Type II had a mean MEPS of 90.7 (N = 52), and Type III had a mean MEPS of 94.2 (N = 27). These results demonstrate the satisfactory outcomes that are attainable after surgical fixation of AMCFs.

There has been a recent focus on determining when repair of the anteromedial coronoid and collateral ligaments is warranted in PMRI cases. Doornberg et al^29^ reported unsatisfactory clinical results after inadequate fixation of AMCFs. Recent findings suggest that the size of the AMCF fragment is an integral component in restoring elbow kinematics. The biomechanical work by Pollock et al^35^ and the computational modeling by Karademir et al^36^ suggested that near-native kinematics of the elbow can be restored with LCL repair without repair of small anteromedial coronoid fragments. In both studies, a ‘small’ fragment was quantified at 2.5 mm of height. In addition, in the presence of a large coronoid fracture fragment (5 mm), repair of the LCL was not sufficient to restore native kinematics. Thus, the authors recommended repair of these large AMCF fragments in addition to the LCL.

**Figure 3:**
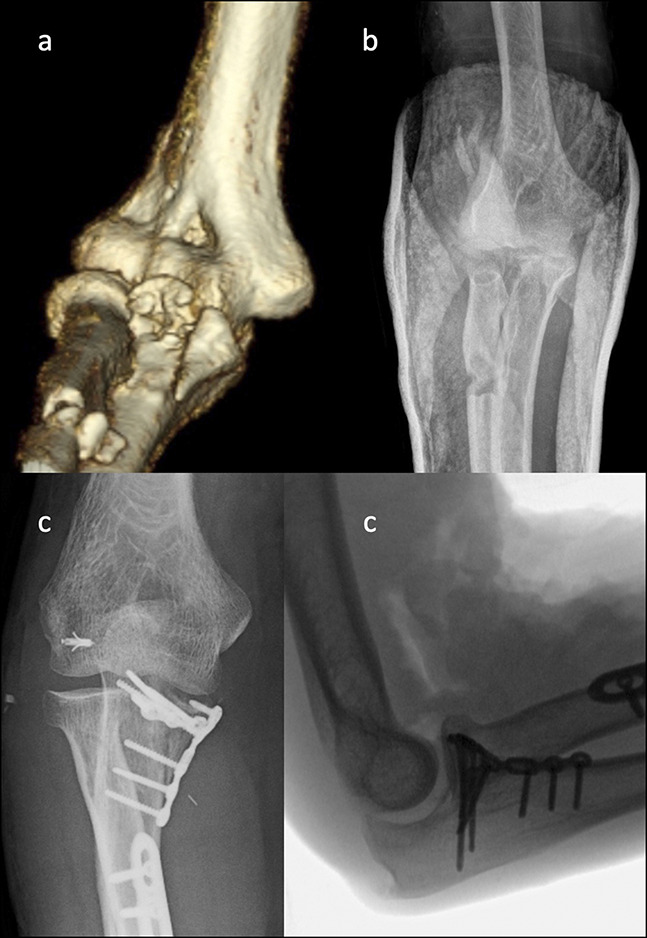
Three-dimensional CT scan (**A**) and radiology image (**B**) demonstrating anteromedial coronoid fracture. Radiographs demonstrating surgical fixation using a coronoid plate and repair of the lateral collateral ligament (**C**).

Stable fixation of AMCFs has been problematic because of the unique forces which act upon this structure.^29^ The anteromedial coronoid must resist compressive loads under varus stress, and it must resist tension loads under valgus stress due to attachment of the anterior band of the MCL at the sublime tubercle. Biomechanical findings for coronoid fracture fixation involving the sublime tubercle (O'Driscoll Type III) concluded that there was greater stability with plate and screw fixation compared with screw fixation.^37^ The superior performance of plating may be because of the buttressing effect of plate fixation, which resists compressive loads under varus and axial stress (Figure 3).

## Joint Stabilization

A stable elbow with a concentric ulnohumeral joint may not be attainable after internal fixation. Adjunct ulnohumeral stabilization can be used when instability persists after internal fixation or to protect a tenuous repair. This can occur in acutely managed cases and especially in those cases that are managed in the subacute or chronic setting. The available options include those which allow motion and those which restrict motion. Ulnohumeral pins can be effective in maintaining joint reduction; however, stiffness may develop because of restricted motion. Ring et al^38^ noted that there are successful treatment options to address stiffness resultant to ulnohumeral transfixion. However, if left untreated, a chronically unstable elbow can lead to irreparable articular injury. Static external fixation is another option that avoids trauma to the joint surface and can be particularly useful in cases with substantial soft-tissue injury. Care must be taken when placing the humeral pins because injury to the radial nerve is a potential complication.

Options for the unstable elbow that provide stability and allow motion include hinged external fixators and novel devices for hinged internal joint fixation and internal bracing treatment. An internal brace uses synthetic tape for ligament augmentation. The literature has demonstrated biomechanical and clinical efficacy for this treatment option. Under simulated PLRI, an internal brace provided stability that was comparable with the intact condition.^39^ Greiner et al^40^ reported on an internal brace after elbow dislocation or fracture-dislocation. Across 17 cases at a median follow-up of 10 months, there were zero cases of recurrent instability; median MEPS was 100; and median DASH score was 18.5. Early postoperative motion was used in all cases. Reports on hinged external fixators have detailed satisfactory outcomes, but problems include effective articulation, pin tract infections, radial nerve injury, and early removal.^41,42^ Rao et al^42^ reported on 20 patients with chronic elbow instability secondary to fracture-dislocation who were treated with static external fixation. At a mean follow-up of nearly 6 years, 95% patients maintained a congruous joint with acceptable function, although 30% patients developed an infection and 25% required surgical release because of joint stiffness. Chamseddine et al^43^ reported a mean MEPS of 90 across 13 cases at a mean follow-up of 7 years for hinged external fixator treatment of unstable elbow injury. The authors reported zero complications related to the pins of the external fixator. Internal joint stabilizers have been described as an internally placed external fixator and are intended to be a transiently implanted device.^44^ Thus, an additional surgical procedure must be considered. Before device implantation, the critical step is identifying the humeral axis of rotation. This axis guides concentric ulnohumeral motion, which avoids articular distraction or compression at extremes of motion. The literature demonstrates low rates of recurrent subluxation with variable rates of complications across a spectrum of elbow instability cases.^45-47^ In a multicenter report, 96% (23/24) cases maintained concentric reduction at 24 to 26 weeks after device removal.^47^ The mean DASH score was 16, and 92% (22/24) patients scored excellent or good on the Broberg-Morrey Functional Score. Currently, the literature has one comparative report using an internal joint stabilizer. Across elbow dislocation or fracture-dislocation, cases that were deemed unstable and treated with an internal joint stabilizer (N = 30) were compared with cases that were deemed stable and not treated with an internal joint stabilizer (N = 34).^48^ One case of recurrent instability in the internal joint stabilizer cohort and two cases of recurrent instability in the cohort that did not use an internal joint stabilizer were noted. When choosing an internal joint stabilizer, device removal is an important consideration. Sochol et al^49^ reported that routine removal was not performed unless by patient request. In their series of 20 cases with an internal joint stabilizer, 6 (30%) were removed, of which 4 did not have implant-associated symptoms. Conversely, Salazar et al^50^ routinely scheduled patients for device removal at 6 to 8 weeks. In their series, cases that required additional time for healing had delayed removal without complications arising because of the delay. The current literature reports a spectrum of time to removal for an internal joint stabilizer. The initial report by Orbay et al^47^ in 2017 described removal at 6 to 8 weeks. In 2020, Pasternack et al reported a mean time to removal of 10.5 weeks,^45^ and in 2022, Salazar et al^50^ reported a mean time to removal of approximately 19 weeks. Continued research will determine the ramifications of retained internal joint stabilizers and the importance of timing of removal.

## Rehabilitation

Postoperative motion is a crucial component of the management algorithm for complex elbow injury. Important considerations include the stable arc of motion and the stability of the fixation construct. These conditions will dictate the parameters of motion in the early postoperative period. Pipicelli and King^51^ recommended controlled mobilization within 1 week after elbow dislocation or fracture-dislocation. Wolff and Hotchkiss^52^ introduced the concept of supine overhead motion to mitigate the joint distraction caused by gravity in the standing position. Biomechanical evidence has validated this concept, with findings of improved elbow stability in the overhead position compared with the standing position.^53,54^ Manocha et al^54^ demonstrated that overhead motion in elbows with MCL/LCL repair was kinematically similar to that of intact elbows. Furthermore, changes in forearm rotation did not affect joint mechanics. Rehabilitation protocols commonly incorporate a progressive strengthening phase, which is important for recovery of dynamic stabilization.

## Case Presentation

### Case 1

**Figure 4:**
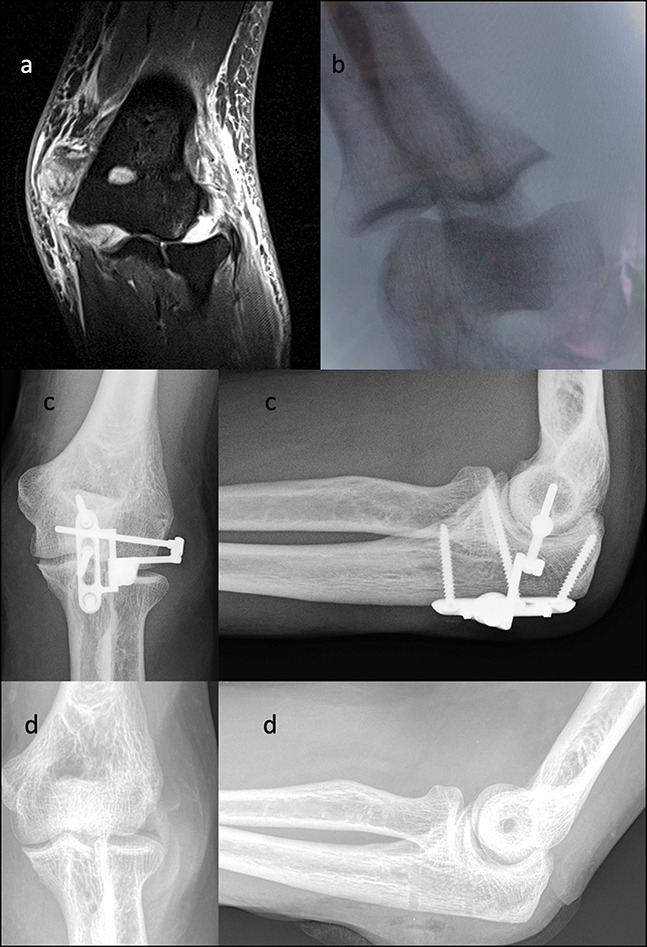
Case 1: Magnetic resonance image (**A**) and fluoroscopy image (**B**) demonstrating gross instability of the elbow with medial and lateral joint widening. Radiographs demonstrating surgical treatment using an internal joint stabilizer to provide stability and maintain a concentric joint (**C**). Twelve-month follow-up radiographs showing a reduced and stable joint (**D**).

A 46-year-old man working on a roof sustained a fall onto his outstretched arm. He presented with 9/10 pain and limited range of motion at the elbow. He was diagnosed with an elbow dislocation, and the joint was reduced in the emergency department. Initial imaging after reduction showed a concentric joint. The results of magnetic resonance imaging (MRI) showed radiocapitellar and ulnohumeral subluxation with evidence of MCL, LCL, common flexor, and common extensor disruption without evidence of fracture (Figure 4, A and B). Surgical treatment occurred 2 weeks after the injury. Intraoperatively, the elbow was grossly unstable and dislocated repeatedly. The lateral ligaments and extensors were avulsed off the distal humerus. The axis pin for the internal joint stabilizer was placed in the event of persistent instability after ligament repair. The avulsed lateral ulnar collateral ligament and lateral extensor insertion were repaired through bone tunnels. After repair, the elbow remained unstable. The decision was made to use the internal joint stabilizer to provide stability and maintain joint congruency (Figure 4, C). There was no period of immobilization, and elbow motion was initiated the day after the surgery under the guidance of the therapist. The device was removed 7 weeks after implantation, and at the 12-month follow-up, the patient had a concentric joint, some heterotopic bone formation, and full unlimited range of motion in supination/pronation and flexion/extension (Figure 4, D).

### Case 2

**Figure 5:**
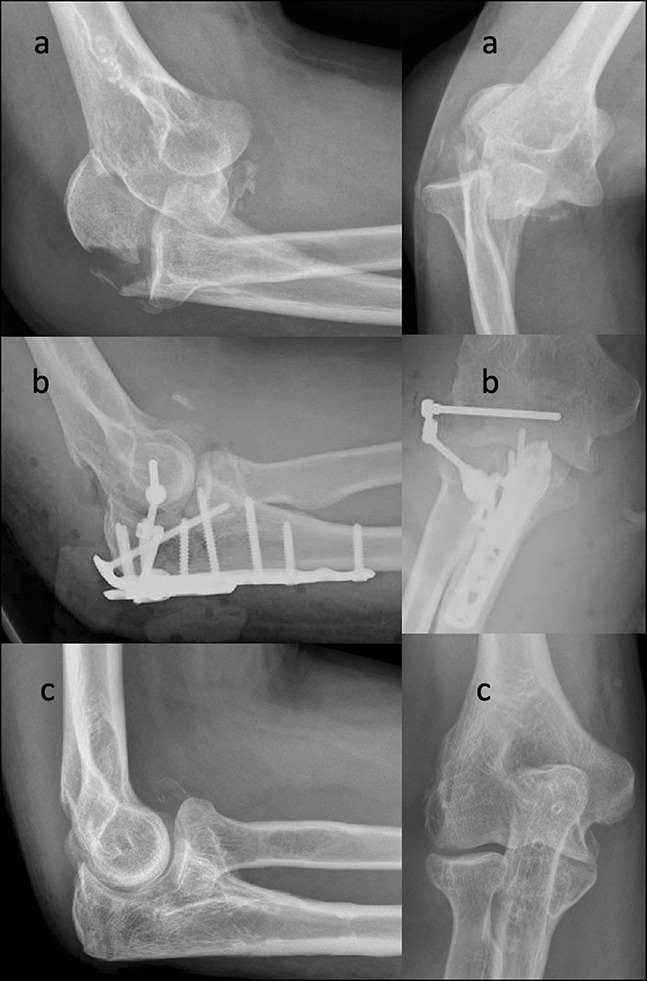
Case 2: Fluoroscopy images demonstrating transolecranon fracture-dislocation (**A**). Radiographs demonstrating surgical fixation using a proximal ulna plate and internal joint stabilizer (**B**). Three-year follow-up radiographs demonstrating that the elbow was stable and the patient had satisfactory function (**C**).

A 58-year-old man presented with a history of recurrent elbow dislocations. He had repeated emergency department visits for a total of six dislocations. All reductions were done in the same emergency department, and all care was within the same hospital system over the course of 3 years. He had six sets of radiographs documenting simple dislocations, followed by concentric reduction. After a fall from a 3-feet height, which resulted in a redislocation, the patient presented for the first time to the orthopaedic department for evaluation. Radiographic investigation showed an elbow dislocation and a complex olecranon fracture (Figure 5, A). The decision was made for surgical management. A posterior approach was used, and the lateral ulnar collateral ligament was noted to be avulsed. After olecranon fixation with a posterior ulna plate, the patient had persistent gross instability with dislocation under minor elbow stress. In this case, an internal joint stabilizer was used to restore stability and joint congruity (Figure 5, B). The elbow was immobilized for 2 days, and then, active range of motion was initiated under the therapy guidance. After 8 weeks, the internal joint stabilizer was removed, and at 2 years, the posterior ulna plate was removed. At 3 years postoperatively, elbow range of motion was 30° to 145°; the elbow was clinical and functionally stable; and the patient experienced no additional dislocations (Figure 5, C).

### Case 3

**Figure 6:**
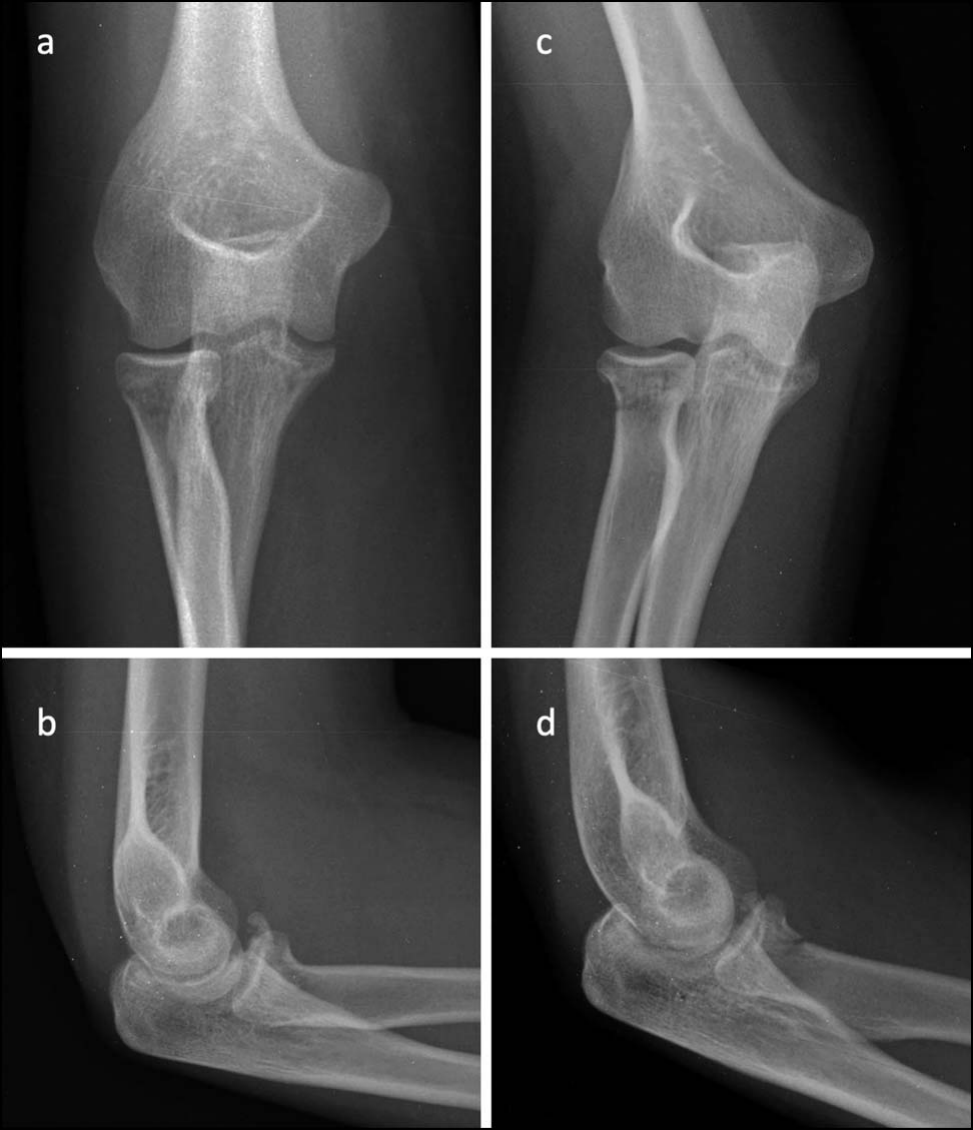
Case 3: Radiology images at the time of injury (**A** and **B**) and after four weeks (**C** and **D**) demonstrating fracture of the anteromedial facet of the coronoid and incomplete fracture of the radial neck.

**Figure 7:**
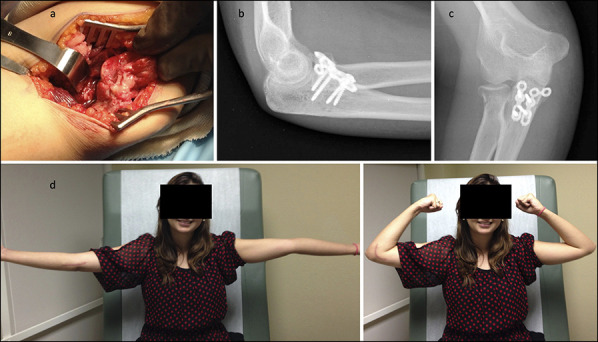
Case 3: Intraoperative image demonstrating fracture of the anteromedial facet of the coronoid under applied varus stress (**A**). Postoperative radiology images after plate and screw fixation of the coronoid fracture (**B** and **C**). Clinical images at eight weeks demonstrating elbow flexion and extension (**D**).

A 22-year-old woman sustained an elbow injury after a fall during in-line skating (Figure 6, A and B). She was managed conservatively at an outside institution and then presented four weeks later with continued pain and loss of elbow motion. Radiographic evaluation revealed an incomplete fracture of the radial neck and an O'Driscoll anteromedial subtype 2 fracture of the coronoid, which may have been missed during initial management (Figure 6, C and D). Intraoperatively, the elbow demonstrated instability in flexion, consistent with PMRI (Figure 7, A). The coronoid was fixed with plates and screws and was considered stable (Figure 7, B and C). At 8 weeks, the patient had a clinically stable elbow, with satisfactory elbow motion of 5° to 145° (Figure 7, D).

## Summary

Historically, our understanding of complex elbow injuries and their surgical management was lacking. The recent literature displays improved outcomes in these cases, in part, because of a more complete comprehension of the injury patterns and fixation methods. Prompt surgical management with stable internal fixation and immediate postoperative mobilization has been a consistent variable across the reports with satisfactory outcomes in acute and chronic cases.
